# Statistics for Next Generation Sequencing – Meeting Report

**DOI:** 10.3389/fgene.2012.00128

**Published:** 2012-07-13

**Authors:** Hao Wu, Michael C. Wu, Degui Zhi, Stephanie A. Santorico, Xiangqin Cui

**Affiliations:** ^1^Department of Biostatistics and Bioinformatics, Emory UniversityAtlanta, GA, USA; ^2^Department of Biostatistics, University of North Carolina at Chapel HillChapel Hill, NC, USA; ^3^Department of Biostatistics, University of Alabama at BirminghamBirmingham, AL, USA; ^4^Department of Mathematical and Statistical Sciences, University of Colorado DenverDenver, CO, USA

The boom of next generation sequencing (NGS) technology and its applications to a wide range of biomedical fields has brought about many computational and statistical challenges. The NHGRI funded small conference “Statistical Analysis for NGS” was held September 26th–27th 2011 in Birmingham, AL, USA, to discuss these statistical challenges and strategies to tackle them. Below, we detail the major themes of the conference.

## Analysis of Rna-Seq Data

Profiling the transcriptome has been a central application of NGS technologies. Since the sequencing technology generates short reads, the first step is to map the reads onto the source genome, genes, and transcripts. Despite development of many algorithms and tools for mapping reads to the reference genomes, accurately mapping RNA-seq reads remains a tough problem due to the complexity of the transcriptome. Thomas Wu from Genentech presented his recent work on mapping RNA-seq sequence reads and gene structure analysis from RNA-seq data in their Genomic Short-read Nucleotide Alignment Program (GSNAP) software package. They use probabilistic models and known splicing information to guide alignment and variant/splicing detection with consideration of all possible combinations of major and minor alleles. Wu also presented GSTRUCT, a pipeline for assembling alignment results to gene structures and predicting isoforms and gene fusion events.

Unlike microarray data, sequencing data were originally thought to be digital and without need for intensive normalization. However, Zhijin Wu from Brown University demonstrated that gene expression data from RNA-seq could suffer from strong biases related to transcript length and GC content. She showed that the biases can be removed by a carefully formulated statistical model which combines a generalized linear model with a quantile normalization procedure^1^.

The clustering of RNA-seq data often relies on heuristic methods used widely in microarray data analysis. Peng Liu from Iowa State University demonstrated that a mixture of negative binomial models for clustering the RNA-seq data together with an Expectation-Maximization (EM) algorithm to obtain the model parameters can improve the performance substantially (R software package *MBCluster.Seq* in CRAN^2^).

In addition to the methodologically focused talks, Ali Mortazavi from UC Irvine presented preliminary results regarding RNA editing events from RNA-seq data generated by the ENCODE project. Mortazavi showed the existence of additional RNA-DNA sequence differences besides SNPs and canonical RNA editing events. He hypothesized that these additional sequence differences are the results of unknown technical artifacts unlike the conclusion from the work of Li et al. (2011).

## Analyzing Chip-Seq Data

ChIP-seq, another major application of NGS technology that analyzes protein-DNA interaction on a genome-wide scale, has provided unprecedented insights into gene regulation and epigenomics. Shirley Liu from Harvard University presented her recent work in modeling transcription and epigenetic regulation from ChIP-seq experiments. She showed that transcription factor (TF) binding changes can be inferred from profiling the dynamic changes of the epigenetic marker H3K4me2 and DNase-sensitive sites in the genome. This strategy overcomes the limitation of antibody availability for individual TFs, which has been a bottleneck for increasing throughput in ChIP-seq experiments.

Method developments for ChIP-seq data analysis have evolved from single experiment peak detection to higher dimensional data integration and pattern recognition. Several talks in the afternoon sessions focused on exploring the regulatory mechanisms of TF binding and histone modifications. Mario Medvedovic from University of Cincinnati presented a statistical framework, termed TREG, for identifying regulatory TF binding by combining ChIP-seq and gene expression data. Hongkai Ji from Johns Hopkins University discussed a method to integrate ChIP-seq data with a large collection of public expression microarray data in the search for TF target genes (*ChIP-PED*). They first compute the correlations between the expression of a TF gene and other genes from 8000+ microarray datasets and then combine the correlations with TF binding locations to detect potential targets.

Epigenetic modification pattern discovery and deciphering its function in gene expression regulation and disease is another exciting direction in ChIP-seq (or MBD-seq) studies. Victor Jin from the Ohio State University Medical Center presented a multivariate hidden Markov model (HMM) to discover distinct DNA methylation patterns under different biological contexts. His results on a breast cancer dataset showed that each subtypes of cancer cell lines have a distinct methylation pattern with specific enrichments in genomic features such as genomic regions, gene expression levels, and functional annotations. Jianrong Wang from Georgia Tech presented an on-going project to cluster genomic regions based on similarities of the patterns of histone modifications. Regions that are closely clustered together are searched for common histone modification patterns. Their analysis on CD4^+^ T cells with over 30 different histone modifications yielded a canonical pattern for active gene promoters.

## Scalable Bioinformatics and Computational Tools

Yingrui Li from BGI (formerly known as Beijing Genomics Institute) provided both a philosophical overview and practical solutions to the problems faced by researchers interested in using sequencing technology to understand how species, individuals, and cells/tissues differ. He described the bottlenecks in the bioinformatics pipeline as breakdowns in both data acquisition and data analysis. To address these bioinformatics challenges, the BGI bioinformatics team has developed a wide range of tools implemented in the Short Oligonucleotide Analysis Package (SOAP). Li reminded participants of the critical role of computer science in turning sophisticated statistical methods into practical bioinformatic tools accessible to biologists.

## Genetic Variant Calling

A central challenge in NGS genomic data analysis is genotype calling. The low allele frequencies for rare variants can be problematic due to low coverage and non-negligible sequencing error rates. Degui Zhi from UAB presented promising strategies and tools for genotype calling for population sequencing. By modifying the HMM emission probabilities, his model can incorporate the fact that individual reads are now able to span multiple sites, particularly as technology progresses and read lengths grow. The strategy shows a substantial genotyping error reduction.

While Zhi's approach is a general algorithm for whole genome variant calling, Danny Challis from Baylor College of Medicine presented an approach that is specifically tailored toward exomic regions, which have different characteristics from other genomic regions and are thought to be biomedically more important. By training a logistic regression model using exon capture data and isolating key features that affect calling errors, Challis developed a powerful predictive model that can discern between true variants and error calls in exomic regions and improve variant calling accuracy (ATLAS2 package).

## Methodology for Rare Variant Analysis

A common hypothesis for the missing heritability in genome-wide association studies is that rare genetic variation may account for a substantial portion of the missing heritability. Several highly inter-related difficulties exist in identifying rare genetic variants associated with disease, including the prohibitive cost of sequencing a large number of individuals and obstacles in association testing. Accordingly, several experts on rare variant analysis presented novel researches on statistical design and analysis strategies for overcoming these challenges.

Standard genetic association methods are underpowered for testing individual rare variants’ association. Region- or gene-based testing, wherein multiple rare variants are aggregated and their cumulative effect on a complex trait or disease evaluated, has become the standard approach for rare variant testing. A veritable plethora of region based tests have been developed under a wide range of assumptions. Suzanne Leal from Baylor College of Medicine, Joshua Sampson from National Cancer Institute, and Michael Wu from UNC Chapel Hill compared many of the commonly applied tests and established connections.

Suzanne Leal systematically assessed the statistical power of many rare variant tests with some assuming that most variants are harmful (unidirectional in effect) and others allowing variants within a region to have bidirectional effects. Across many scenarios, the methods performed similarly with the unidirectional methods tending to be more powerful, though there were also settings in which the power was somewhat higher for methods accommodating bidirectional effects.

Joshua Sampson and Michael Wu sought to make connections among many of the existing methods. Sampson showed that the majority of statistics that are currently available for testing associations with groups of rare variants can be rewritten as a simple weighted sum of single variant statistics and their cross products. He demonstrated how to identify those statistics that perform well given a set of genetic characteristics. In a similar line of work, Wu showed that as simple weighted score statistics, many key rare variant tests reduce to special cases of Kernel Association test. Wu developed a pragmatic strategy that involves applying several candidate rare variant tests, taking the minimum *p*-value, and then correcting for multiple comparisons via permutation. The new strategy has high power across a wide range of scenarios.

## Study Design for Large Scale Sequencing Experiments

Currently, even exome scale sequencing is expensive and results in limited sample sizes and hence limited power to detect rare variants with modest effects. A number of powerful design strategies can be used to ameliorate these issues. Hongyu Zhao from Yale considered the use of pooled DNA designs in which the DNA of several cases or several controls is pooled and jointly sequenced. He showed that the optimal number of individuals in a pool, with regard to detection probability is a function of minor allele frequency (MAF), read depth, and detection threshold. Interestingly, the optimal number of individuals did not differ much across MAF, but the total number of pools necessary to detect lower MAF variants was greater. An alternative cost saving strategy for population based rare variant analysis is the Exome Chip as Susanne Leal pointed out. This chip focuses mostly on the exomic regions to allow for the capture of many of the variants that are more likely to be functionally relevant. In addition to exome variants, the chip also includes SNPs for population structure, probes for HLA tags, fingerprinting SNPs, and a range of other variants of primary interest. However, Leal noted that the chips will have limited power in studies of Mendelian traits or traits with high degree of allelic heterogeneity and that it is unclear how well the chips will work for non-European populations. Nevertheless, they can provide a means for analysis of exomic regions at a fraction of the cost of exome sequencing.

## Methodology for Analysis in Cancer Genomes

*Cancer genome sequence data analysis post special* challenges due to the massive genomic changes. Benjamin Raphael from Brown University and Stanley Pounds from St. Jude Children's Research Hospital offered some novel strategies for the analysis of tumor sequencing data. Raphael discussed a method for identifying driver somatic mutations from the background of passenger mutations in tumor progression. His strategies involve identifying subgraphs that are recurrently mutated across multiple patients using a heat diffusion strategy for defining subnetworks, which offer improvements over standard individual mutation analysis. Pounds examined the analysis of copy number variations (CNVs). The standard steps in the analysis pipeline for CNVs are often (1) mapping of reads, (2) normalization and adjustment for aneuploidy, (3) comparison of counts between tumor and normal tissues, and (4) segmentation to identify change points. Pounds re-ordered the steps, choosing to compare raw counts between tumor and normal immediately following mapping and then moving the adjustment for ploidy to the end of the analysis pipeline, after segmentation. This new order improved determination of copy number alterations due to reduced noise.

After 2 days of enthusiastic discussion on the statistical challenges and opportunities associated with NGS technologies, participants left with more questions than answers. These questions will require the whole community to work tirelessly for the next several years to come up with mature statistical and computational methods for NGS data analysis.
